# Supplementary material for: Sorting at embryonic boundaries requires high heterotypic interfacial tension
Source: Nat Commun. 2017 Jul 31;8:157. doi: 10.1038/s41467-017-00146-x (PMC5537356; doi:10.1038/s41467-017-00146-x)
Supplement: Supplementary file 2 — Supplementary Software 1 [file 41467_2017_146_MOESM2_ESM.zip › PottsModel/SrcPottsModel/doc/model/class-use/Constants.html]

Uses of Class model.Constants


JavaScript is disabled on your browser.


Skip navigation links


- Overview
- Package
- Class
- Use
- Tree
- Deprecated
- Index
- Help

- Prev
- Next

- Frames
- No Frames

- All Classes

## Uses of Class model.Constants

- Packages that use Constants

  | Package | Description |
  | --- | --- |
  |  |  |
  | --- | --- |
  | engine |  |
  | gui |  |
  | model |  |
  | simulations |  |
- - ### Uses of Constants in engine

    Methods in engine that return Constants

    | Modifier and Type | Method and Description |
    | --- | --- |
    |  |  |
    | --- | --- |
    | `abstract Constants[]` | Simulation.`getConstants()` |
    | `Constants` | PottsEngine.`getConstants()` |

    Constructors in engine with parameters of type Constants

    Constructor and Description

    `PottsEngine(Constants pConstants)`

    `PottsEngine(Constants[] pConstants)`

    `PottsEngine(Constants pConstants,
    Lattice pLattice)`
  - ### Uses of Constants in gui

    Constructors in gui with parameters of type Constants

    Constructor and Description

    `ConfigurationPanel(Constants pConstants)`

    `PottsCanvas(int pPottsWidth,
    int pPottsHeight,
    Constants c)`

    Constructs a Cartesian grid that can carry (pPottsWidth x pPottsHeight)
    PottsPixels a.k.a Spins.

    `SnapshotManager(int width,
    int height,
    Constants c)`

    Constructs a Cartesian grid that can carry (pPottsWidth x pPottsHeight)
    PottsPixels a.k.a Spins.
  - ### Uses of Constants in model

    Fields in model declared as Constants

    | Modifier and Type | Field and Description |
    | --- | --- |
    |  |  |
    | --- | --- |
    | `Constants` | Cell.`aConstants` |

    Methods in model that return Constants

    | Modifier and Type | Method and Description |
    | --- | --- |
    |  |  |
    | --- | --- |
    | `Constants` | Pixel.`getConstants()` |
    | `Constants` | Lattice.`getConstants()` |
    | `Constants` | KnowsConstants.`getConstants()` |
    | `Constants` | Cell.`getConstants()` |

    Methods in model with parameters of type Constants

    | Modifier and Type | Method and Description |
    | --- | --- |
    |  |  |
    | --- | --- |
    | `static Lattice` | Lattice.`inverse(Constants pConstants)` |

    Constructors in model with parameters of type Constants

    Constructor and Description

    `EnergyManager(Constants c,
    Constants.Model m,
    PottsEngine.StateVariables s)`

    `Lattice(Constants pConstants)`

    `Lattice(Constants pConstants,
    int[][][] rawLattice)`

    Initialize grid with specified pixel IDs.
  - ### Uses of Constants in simulations

    Methods in simulations that return Constants

    | Modifier and Type | Method and Description |
    | --- | --- |
    |  |  |
    | --- | --- |
    | `Constants[]` | SingleCell.`getConstants()` |
    | `Constants[]` | NegativeControl.`getConstants()` |
    | `Constants[]` | GranerGlazierSimulation.`getConstants()` |
    | `Constants[]` | GenerateRandomLattices.`getConstants()` |
    | `Constants[]` | FourByFourSimulation.`getConstants()` |
    | `Constants[]` | EndoSimulation.`getConstants()` |
    | `Constants[]` | DITHSimulation.`getConstants()` |
    | `Constants[]` | DefaultSimulation.`getConstants()` |
    | `Constants[]` | BoundarySimulation.`getConstants()` |

Skip navigation links


- Overview
- Package
- Class
- Use
- Tree
- Deprecated
- Index
- Help

- Prev
- Next

- Frames
- No Frames

- All Classes
